# RETRACTED: Alhakamy et al. Thymoquinone-Loaded Soy-Phospholipid-Based Phytosomes Exhibit Anticancer Potential against Human Lung Cancer Cells. *Pharmaceutics* 2020, *12,* 761

**DOI:** 10.3390/pharmaceutics16020156

**Published:** 2024-01-23

**Authors:** Nabil A. Alhakamy, Shaimaa M. Badr-Eldin, Usama A. Fahmy, Nabil K. Alruwaili, Zuhier A. Awan, Giuseppe Caruso, Mohamed A. Alfaleh, Ahmed L. Alaofi, Faris O Arif, Osama A. A. Ahmed, Adel F. Alghaith

**Affiliations:** 1 Department of Pharmaceutics, Faculty of Pharmacy, King Abdulaziz University, Jeddah 21589, Saudi Arabia; nalhakamy@kau.edu.sa (N.A.A.); smbali@kau.edu.sa (S.M.B.-E.); oaahmed@kau.edu.sa (O.A.A.A.); 2 Advanced Drug Delivery Research Group, Faculty of Pharmacy, King Abdulaziz University, Jeddah 21589, Saudi Arabia; 3 Center of Excellence for Drug Research and Pharmaceutical Industries, King Abdulaziz University, Jeddah 21589, Saudi Arabia; 4 Department of Pharmaceutics and Industrial Pharmacy, Faculty of Pharmacy, Cairo University, Cairo 11562, Egypt; 5 Department of Pharmaceutics, College of Pharmacy, Jouf University, Sakaka, Al-Jouf 2014, Saudi Arabia; nkalruwaili@ju.edu.sa; 6 Department of Clinical Biochemistry, Faculty of Medicine, King Abdulaziz University, Jeddah 21589, Saudi Arabia; zawan@kau.edu.sa; 7 Oasi Research Institute—IRCCS, Via Conte Ruggero, 73, 94018 Troina (EN), Italy; 8 Department of Natural Products and Alternative Medicine, Faculty of Pharmacy, King Abdulaziz University, Jeddah 21589, Saudi Arabia; maalfaleh@kau.edu.sa; 9 Department of Pharmaceutics, College of Pharmacy, King Saud University, P.O. Box 2457, Riyadh 11451, Saudi Arabia; ahmedofi@ksu.edu.sa (A.L.A.); afalghaith@ksu.edu.sa (A.F.A.); 10 General Surgery KAUH, King Abdulaziz University Hospital, Jeddah 21589, Saudi Arabia; farif0001@stu.kau.edu.sa

The journal retracts the article, “Thymoquinone-Loaded Soy-Phospholipid-Based Phytosomes Exhibit Anticancer Potential against Human Lung Cancer Cells” [1] cited above. 

Following publication, concerns were brought to the attention of the publisher regarding duplicated images across other publications [2,3,4,5,6,7], representing different experimental conditions.

Adhering to our complaint procedure, an investigation was conducted that confirmed that Figure 7B published in [1] is a duplicate of Figure 8B of [2], Figure 7B of [3], Figure 7B of [4], Figure 7C of [5], Figure 4C of [6] and Figure S2 of [7]. 

While the authors fully cooperated with the Editorial Office during the investigation, they were unable to satisfactorily explain the overlap of figures and meet the required quality standards of original images in order to consider a correction as per the journal’s original image requirements policy (https://www.mdpi.com/journal/pharmaceutics/instruc-tions#oriimages). As a result, the Editorial Board and Editor-in-Chief were unable to confirm the reliability of the findings and subsequently decided to retract this paper.

This retraction was approved by the Editor-in-Chief of the journal *Pharmaceutics*. 

The authors did not agree to this retraction. 
